# Supplementary material for: Determinants of Complementary Feeding Indicators: A Secondary Analysis of Thailand Multiple Indicators Cluster Survey 2019
Source: Nutrients. 2022 Oct 18;14(20):4370. doi: 10.3390/nu14204370 (PMC9610694; doi:10.3390/nu14204370)
Supplement: Supplementary file 1 [file nutrients-14-04370-s001.zip › nutrients-1925964-supplementary.pdf]

## Supplementary Materials

Table S1. Comparison indicators in 2008 and 2021 and their key revision

| Indicators | 2008 | 2021 | Key revision |
|---|---|---|---|
| Introduction of solid, semi-solid or soft foods 6–8 months (ISSSF) | / | / | Calculation is now based on the food list question rather than the frequency of feeding question |
| Minimum dietary diversity 6–23 months (MDD) | / | / | Breast milk added as an eighth food group and cut-off for minimum increased to five food groups |
| Minimum meal frequency 6–23 months (MMF) | / | / | At least one non-milk feeding is required to meet minimum for non-breastfed children while the previous definition allowed children to achieve the minimum with milk feeds only. |
| Minimum acceptable diet 6–23 months (MAD) | / | / | |
| Consumption of iron-rich or iron-fortified foods | / | x | |
| Minimum milk feeding frequency for non-breastfed children 6–23 months (MMFF) | / | / | |
| Egg and/or flesh food consumption 6–23 months (EFF) | x | / | |
| Sweet beverage consumption 6–23 months (SwB) | x | / | |
| Unhealthy food consumption 6–23 months (UFC) | x | / | |
| Zero vegetable or fruit consumption 6–23 months (ZVF) | x | / | |

Table S2. Factors associated with complementary feeding indicators in children who lived with their mothers, the univariate logistic regression analysis

| | ISSSF | | MDD | | MMF | | MMFF | | MAD | | EFF | | SWB | | UFC | | ZVF | |
|---|---|---|---|---|---|---|---|---|---|---|---|---|---|---|---|---|---|---|
| | **OR** | **95% CI** | **OR** | **95% CI** | **OR** | **95% CI** | **OR** | **95% CI** | **OR** | **95% CI** | **OR** | **95% CI** | **OR** | **95% CI** | **OR** | **95% CI** | **OR** | **95% CI** |
| **Gender of children (v male)** | | | | | | | | | | | | | | | | | | |
| Female | 0.59 | 0.30, 1.16 | 1.07 | 0.92, 1.25 | 0.83 | 0.60, 1.15 | 0.82 | 0.52, 1.28 | 1.07 | 0.92, 1.24 | 0.89 | 0.69, 1.14 | 1.08 | 0.93, 1.26 | 1.01 | 0.82, 1.25 | 0.97 | 0.80, 1.18 |
| **Age of children (months) (v 6-8)** | | | | | | | | | | | | | | | | | | |
| 9-11 | | | 2.33** | 1.81, 3.01 | 4.09** | 2.42, 6.91 | 4.89 | 0.98, 24.53 | 2.47** | 1.91, 3.19 | 3.37** | 2.38, 4.75 | 1.31* | 1.01, 1.70 | 1.68* | 1.06, 2.65 | 0.33** | 0.24, 0.45 |
| 12-17 | | | 3.81** | 3.01, 4.82 | 4.40** | 2.80, 6.92 | 1.17 | 0.46, 2.99 | 3.26** | 2.58, 4.12 | 7.43** | 5.21, 10.59 | 1.41** | 1.12, 1.78 | 2.52** | 1.67, 3.81 | 0.29** | 0.22, 0.38 |
| 18-23 | | | 3.45** | 2.74, 4.33 | 3.40** | 2.26, 5.11 | 0.50 | 0.21, 1.19 | 3.25** | 2.59, 4.09 | 10.80** | 7.33, 15.92 | 1.46** | 1.16, 1.84 | 3.39** | 2.25, 5.12 | 0.26** | 0.20, 0.34 |
| **Age of mothers or caregivers (years) (v >35)** | | | | | | | | | | | | | | | | | | |
| 20-35 | 0.56 | 0.21, 1.48 | 0.85 | 0.70, 1.03 | 1.05 | 0.70, 1.57 | 0.59 | 0.30, 1.17 | 0.85 | 0.70, 1.02 | 0.80 | 0.57, 1.11 | 1.03 | 0.86, 1.25 | 1.14 | 0.87, 1.48 | 1.00 | 0.79, 1.28 |
| 15-19 | 1.89 | 0.21, 16.83 | 0.93 | 0.67, 1.30 | 1.12 | 0.54, 2.31 | 0.59 | 0.21, 1.65 | 0.92 | 0.66, 1.29 | 0.91 | 0.51, 1.62 | 0.96 | 0.69, 1.34 | 1.10 | 0.70, 1.74 | 1.04 | 0.69, 1.59 |
| **Language of mothers or caregivers(v Thai)** | | | | | | | | | | | | | | | | | | |
| Non-Thai | 1.87 | 0.56, 6.29 | 1.09 | 0.87, 1.37 | 0.77 | 0.50, 1.20 | 0.43** | 0.25, 0.76 | 0.98 | 0.78, 1.22 | 1.28 | 0.85, 1.90 | 0.74* | 0.59, 0.91 | 0.65** | 0.48, 0.87 | 0.81 | 0.60, 1.10 |
| **Education level of mothers or caregivers (v Kindergarten and Primary)** | | | | | | | | | | | | | | | | | | |
| High school and above | 1.45 | 0.61, 3.47 | 1.52** | 1.22, 1.88 | 1.58* | 1.03, 2.41 | 1.60 | 0.85, 3.03 | 1.57** | 1.26, 1.95 | 1.69** | 1.21, 2.35 | 1.61** | 1.30, 2.01 | 1.08 | 0.80, 1.47 | 0.62** | 0.47, 0.80 |
| **Wealth index quintiles (v poorest)** | | | | | | | | | | | | | | | | | | |
| Second | 2.48 | 0.83, 7.44 | 1.34* | 1.06, 1.68 | 1.15 | 0.69, 1.90 | 1.43 | 0.75, 2.74 | 1.33* | 1.06, 1.67 | 1.14 | 0.78, 1.67 | 1.26* | 1.01, 1.58 | 0.92 | 0.66, 1.30 | 0.82 | 0.62, 1.08 |
| Middle | 3.17 | 0.97, 10.33 | 1.41** | 1.12, 1.77 | 1.22 | 0.73, 2.03 | 1.30 | 0.70, 2.41 | 1.33* | 1.06, 1.67 | 1.19 | 0.82, 1.73 | 1.61** | 1.28, 2.01 | 0.70* | 0.50, 0.97 | 0.66** | 0.50, 0.88 |
| Fourth | 1.09 | 0.43, 2.78 | 1.48** | 1.17, 1.87 | 0.88 | 0.55, 1.43 | 2.05* | 1.03, 4.06 | 1.41** | 1.12, 1.79 | 1.15 | 0.78, 1.68 | 2.41** | 1.88, 3.09 | 0.58** | 0.41, 0.81 | 0.56** | 0.41, 0.75 |
| Richest | 0.82 | 0.32, 2.10 | 1.97** | 1.53, 2.54 | 1.20 | 0.70, 2.06 | 4.18** | 1.67, 10.44 | 2.01** | 1.56, 2.59 | 1.51 | 0.98, 2.32 | 1.77** | 1.38, 2.27 | 0.56** | 0.39, 0.81 | 0.44** | 0.31, 0.62 |
| **Area (v urban)** | | | | | | | | | | | | | | | | | | |
| Rural | 1.07 | 0.53, 2.15 | 1.00 | 0.85, 1.17 | 1.04 | 0.74, 1.46 | 0.68 | 0.42, 1.11 | 1.00 | 0.85, 1.17 | 1.05 | 0.81, 1.37 | 0.85 | 0.73, 1.00 | 1.12 | 0.90, 1.39 | 1.11 | 0.90, 1.36 |
| **Region (v Bangkok and Central)** | | | | | | | | | | | | | | | | | | |
| North | 1.49 | 0.52, 4.28 | 0.72* | 0.57, 0.90 | 1.16 | 0.68, 1.97 | 0.56 | 0.30, 1.06 | 0.66** | 0.52, 0.83 | 1.09 | 0.76, 1.56 | 0.65** | 0.52, 0.82 | 0.85 | 0.60, 1.21 | 1.04 | 0.77, 1.41 |
| Northeast | 1.50 | 0.63, 3.55 | 1.02 | 0.84, 1.24 | 0.93 | 0.61, 1.40 | 0.91 | 0.49, 1.68 | 1.02 | 0.84, 1.24 | 1.75** | 1.24, 2.45 | 0.78* | 0.64, 0.95 | 1.17 | 0.86, 1.59 | 1.24 | 0.97, 1.59 |
| South | 1.33 | 0.56, 3.16 | 1.31* | 1.06, 1.60 | 1.06 | 0.69, 1.64 | 0.78 | 0.42, 1.42 | 1.20 | 0.98, 1.46 | 1.79** | 1.27, 2.52 | 0.86 | 0.70, 1.05 | 0.45** | 0.35, 0.59 | 0.85 | 0.65, 1.11 |
| **Current breastfeeding (v No)** | | | | | | | | | | | | | | | | | | |

| | ISSSF | | MDD | | MMF | | MMFF | | MAD | | EFF | | SWB | | UFC | | ZVF | |
|---|---|---|---|---|---|---|---|---|---|---|---|---|---|---|---|---|---|---|
| | 0.31* | 0.13, 0.72 | 1.00 | 0.85, 1.17 | 0.10** | 0.07, 0.16 | | | 0.76** | 0.65, 0.88 | 0.40** | 0.31, 0.52 | 0.17** | 0.15, 0.20 | 0.75* | 0.60, 0.95 | 1.37** | 1.12, 1.67 |

*P < 0.05; **P < 0.01, N/A = not available; ****reported receiving any breastmilk in the past 24 hours

ISSF = Introduction of solid, semi-solid or soft foods 6-8 months; MDD = Minimum dietary diversity 6–23 months; MMF = Minimum meal frequency 6–23 months; MMFF = Minimum milk feeding frequency for non-breastfed children 6–23 months; MAD = Minimum acceptable diet 6–23 months; EFF = Egg and/or flesh food consumption 6–23 months; SwB = Sweet beverage consumption 6–23 months; UFC = Unhealthy food consumption 6–23 months; ZVF = Zero vegetable or fruit consumption 6–23 months; OR = Odds ratio; 95%CI = 95% confidence interval

## Table S3. Factors associated with complementary feeding indicators in children who lived with others, the univariate logistic regression analysis

| | ISSSF | | MDD | | MMF | | MMFF | | MAD | | EFF | | SWB | | UFC | | ZVF | |
|---|---|---|---|---|---|---|---|---|---|---|---|---|---|---|---|---|---|---|
| | **OR** | **95% CI** | **OR** | **95% CI** | **OR** | **95% CI** | **OR** | **95% CI** | **OR** | **95% CI** | **OR** | **95% CI** | **OR** | **95% CI** | **OR** | **95% CI** | **OR** | **95% CI** |
| **Gender of children (v male)** | | | | | | | | | | | | | | | | | | |
| Female | 1.02 | 0.20, 5.36 | 1.17 | 0.90, 1.52 | 1.08 | 0.49, 2.36 | 1.03 | 0.56, 1.89 | 1.19 | 0.92, 1.55 | 1.10 | 0.72, 1.69 | 1.71** | 1.23, 2.37 | 0.79 | 0.52, 1.22 | 0.97 | 0.71, 1.33 |
| **Age of children (months) (v 6-8)** | | | | | | | | | | | | | | | | | | |
| 9-11 | | | 3.23** | 1.86, 5.60 | 0.79 | 0.19, 3.25 | 0.95 | 0.30, 3.00 | 3.75** | 2.11, 6.67 | 3.52** | 1.88, 6.60 | 0.95 | 0.44, 2.05 | 1.68 | 0.47, 6.09 | 0.50* | 0.29, 0.88 |
| 12-17 | | | 4.25** | 2.58, 6.98 | 10.67* | 1.09, 104.00 | 2.55 | 0.79, 8.27 | 4.97** | 2.94, 8.42 | 7.94** | 4.33, 14.57 | 1.04 | 0.52, 2.09 | 1.81 | 0.56, 5.80 | 0.31** | 0.19, 0.51 |
| 18-23 | | | 4.23** | 2.61, 6.85 | 0.84 | 0.24, 2.95 | 0.93 | 0.34, 2.52 | 4.68** | 2.80, 7.81 | 13.58** | 7.12, 25.89 | 0.38** | 0.20, 0.73 | 2.61 | 0.82, 8.33 | 0.29** | 0.18, 0.48 |
| **Age of mothers or caregivers (years) (v >35)** | | | | | | | | | | | | | | | | | | |
| 20-35 | N/A | | 1.34 | 0.39, 4.56 | 0.37 | 0.04, 3.23 | 0.12* | 0.03, 0.53 | 0.89 | 0.26, 3.00 | 0.49 | 0.10, 2.36 | 0.59 | 0.15, 2.29 | 0.98 | 0.10, 9.75 | 1.19 | 0.31, 4.55 |
| 15-19 | | | | | | | | | | | | | | | | | | |
| **Language of mothers or caregivers(v Thai)** | | | | | | | | | | | | | | | | | | |
| Non-Thai | 0.48 | 0.05, 4.32 | 1.18 | 0.88, 1.58 | 1.50 | 0.66, 3.41 | 1.06 | 0.53, 2.09 | 1.11 | 0.82, 1.49 | 0.81 | 0.49, 1.34 | 0.77 | 0.52, 1.12 | 1.07 | 0.67, 1.72 | 0.86 | 0.61, 1.23 |
| **Education level of mothers or caregivers (v Kindergarten and Primary)** | | | | | | | | | | | | | | | | | | |
| High school and above | N/A | | 1.25 | 0.71, 2.21 | 0.88 | 0.22, 3.51 | 0.77 | 0.22, 2.64 | 1.35 | 0.76, 2.39 | 1.40 | 0.50, 3.92 | 1.03 | 0.49, 2.20 | 0.51 | 0.22, 1.19 | 0.59 | 0.29, 1.23 |

| | ISSSF | | MDD | | MMF | | MMFF | | MAD | | EFF | | SWB | | UFC | | ZVF | |
|---|---|---|---|---|---|---|---|---|---|---|---|---|---|---|---|---|---|---|
| **Wealth index quintiles (v poorest)** | | | | | | | | | | | | | | | | | | |
| Second | 1.47 | 0.12, 17.21 | 1.67** | 1.19, 2.34 | 1.37 | 0.48, 3.92 | 1.09 | 0.54, 2.22 | 1.69** | 1.20, 2.38 | 1.16 | 0.67, 2.00 | 1.16 | 0.76, 1.78 | 1.07 | 0.59, 1.94 | 0.73 | 0.49, 1.08 |
| Middle | 1.40 | 0.12, 16.46 | 1.57* | 1.08, 2.27 | 0.98 | 0.34, 2.80 | 1.97 | 0.77, 5.06 | 1.61* | 1.10, 2.34 | 0.97 | 0.55, 1.72 | 0.81 | 0.53, 1.26 | 0.63 | 0.35, 1.13 | 0.61* | 0.39, 0.96 |
| Fourth | N/A | | 1.39 | 0.91, 2.10 | 2.04 | 0.43, 9.60 | 4.00 | 0.91, 17.51 | 1.52 | 0.99, 2.32 | 1.90 | 0.85, 4.24 | 0.86 | 0.52, 1.42 | 0.55 | 0.29, 1.04 | 0.62 | 0.37, 1.04 |
| Richest | 0.03* | 0.00, 0.55 | 1.92* | 1.03, 3.59 | 0.54 | 0.14, 2.06 | 1.02 | 0.29, 3.61 | 1.99* | 1.07, 3.70 | 1.18 | 0.44, 3.17 | 1.60 | 0.69, 3.71 | 0.93 | 0.32, 2.71 | 0.52 | 0.23, 1.15 |
| **Area (v urban)** | | | | | | | | | | | | | | | | | | |
| Rural | 6.08* | 1.10, 33.73 | 0.95 | 0.70, 1.29 | 0.60 | 0.21, 1.78 | 0.99 | 0.48, 2.05 | 0.98 | 0.72, 1.34 | 0.59 | 0.33, 1.05 | 0.86 | 0.59, 1.27 | 1.09 | 0.67, 1.78 | 1.41 | 0.95, 2.09 |
| **Region (v Bangkok and Central)** | | | | | | | | | | | | | | | | | | |
| North | 0.65 | 0.06, 7.64 | 1.13 | 0.72, 1.78 | 1.48 | 0.29, 7.50 | 1.15 | 0.39, 3.40 | 1.16 | 0.73, 1.86 | 1.31 | 0.68, 2.55 | 1.41 | 0.80, 2.48 | 0.67 | 0.31, 1.46 | 0.64 | 0.37, 1.11 |
| Northeast | 6.65 | 0.66, 67.43 | 1.97** | 1.43, 2.70 | 0.89 | 0.35, 2.29 | 1.25 | 0.60, 2.61 | 2.15** | 1.55, 2.97 | 2.31** | 1.43, 3.73 | 1.58* | 1.08, 2.30 | 0.71 | 0.41, 1.22 | 0.55** | 0.38, 0.79 |
| South | 0.78 | 0.07, 8.93 | 2.95** | 1.74, 5.00 | 2.68 | 0.32, 22.62 | 0.99 | 0.33, 2.94 | 2.75** | 1.63, 4.64 | 4.55* | 1.57, 13.15 | 1.40 | 0.78, 2.52 | 0.52 | 0.26, 1.07 | 0.34** | 0.17, 0.65 |
| **Current breastfeeding (v No)** | | | | | | | | | | | | | | | | | | |
| | 0.26 | 0.02, 2.74 | 0.92 | 0.41, 2.04 | 0.06** | 0.02, 0.17 | | | 0.45 | 0.19, 1.09 | 0.36* | 0.14, 0.93 | 0.28** | 0.12, 0.61 | 1.21 | 0.25, 5.80 | 1.48 | 0.61, 3.58 |

*P < 0.05; **P < 0.01, N/A = not available; ****reported receiving any breastmilk in the past 24 hours

ISSF = Introduction of solid, semi-solid or soft foods 6-8 months; MDD = Minimum dietary diversity 6–23 months; MMF = Minimum meal frequency 6–23 months; MMFF = Minimum milk feeding frequency for non-breastfed children 6–23 months; MAD = Minimum acceptable diet 6–23 months; EFF = Egg and/or flesh food consumption 6–23 months; SwB = Sweet beverage consumption 6–23 months; UFC = Unhealthy food consumption 6–23 months; ZVF = Zero vegetable or fruit consumption 6–23 months; OR = Odds ratio; 95%CI = 95% confidence interval
